# Exploring the 4D printing linked bio-smart materials in dentistry: a concise overview

**DOI:** 10.3389/fdmed.2025.1558382

**Published:** 2025-04-16

**Authors:** Maajida Aafreen M, Priyanka K. Cholan, Paavai Ilango, Harinath Parthasarathy, Anupama Tadepalli, Lakshmi Ramachandran

**Affiliations:** ^1^ Department of Periodontology and Oral Implantology, SRM Dental College and Hospitals, Chennai, Tamil Nadu, India; ^2^ Department of Periodontology and Oral Implantology, Priyadarshini Dental College, Chennai, Tamil Nadu, India

**Keywords:** 4D printing, bio-smart materials, smart materials, hydrogels, smart polymers, responsive

## Abstract

4D printing advances traditional 3D printing by incorporating the dimension of time, enabling stimuli-responsive shape or behavior changes. Bio-smart materials, crucial to this technology, enable programmable transformations with significant potential in biomechanics and dentistry. This review explores the use of smart materials and stimuli in 4D printing, emphasizing dental applications.A comprehensive search across EMBASE, Web of Science, MEDLINE, Cochrane Library and clinical trial registries identified 154 articles on 4D printing technologies, biomaterials, and stimuli relevant to dental applications. Of these, 84 were pertinent to the review's objective, with 25 specifically focused on 4D printing and various smart materials. The review highlights biomaterials engineered for programmable responses, such as shape memory polymers, shape memory elastomers, responsive inks, and hydrogels. These materials enable the creation of structures that can adapt, self-assemble, or respond to stimuli like temperature, moisture, or pH levels. In dentistry, these capabilities show potential for applications in orthodontics, implants, and tissue engineering.The integration of 4D printing and bio-smart materials has the potential to transform dentistry by creating adaptive, time-responsive structures. This technology enables personalized, precise, and minimally invasive treatments, addressing complex biomechanical challenges in dental care.

## 1 Introduction

4D printing is currently defined as the ability of a 3D printed object to change its shape, properties, and functions over time when exposed to specific triggers such as heat, water, light, pH, and more (1). The fourth dimension in the 4D printing process is called “Time,” and it allows printed objects to change, adapt, or self-assemble into complex structures resulting in the shift from static to dynamic, in contrast to three-dimensional (3D) printing, which often produces static constructs with fixed shapes and functions (2). This 4D printing involves bio-smart materials that fold themselves and experience spontaneous shape change in response to thermal and moisture fluctuations. This is based on using 3D printing to create objects from multiple materials, which are then selectively cured through photo-curing to give them the ability to move (3). Evaluating the strain properties of individual components in the printed model and subjecting them to a controllable pattern can help to determine the transformation mechanism (4). Furthermore, single-material items that can alter their form and principal stress-bearing point to adjust their internal tension may now be produced using 4D printing technology (5).

Smart materials are engineered substances capable of undergoing significant changes in their properties in response to external stimuli such as mechanical stress, temperature, moisture, pH, and electric or magnetic fields (6). The advent of these materials has enabled their application in areas such as shape recovery, sensors, and actuators. A pivotal aspect of 4D printing is the ability of printed objects to morph their shape post-fabrication, triggered by various external stimuli, leading to expansion, shrinkage, or folding. The dynamics of these transformations are governed by different principles, depending on whether the objects are composed of a single smart material or bilayer structures with varying properties and inhomogeneities (7). Smart materials are pivotal to advancing 4D printing research. Although numerous smart materials are being developed, not all are suitable for 4D printing. Additionally, smart materials need not exhibit shape-changing properties to contribute significantly to 4D printing research. Materials that can alter their color, hardness, or transparency hold potential for applications in camouflage technology, user signaling, foreign substance detection, and biomedical fields (8).

With 4D printing, materials can be designed to alter their shape over time or space, allowing for precise control of subtle adjustments beneficial in biomechanical applications. 4D printing and smart materials are transforming the field of medicine by enabling researchers and practitioners to create medical devices and implants that are not only more effective but also more adaptable to the patient's body. With the ability to embed smart materials into the design of medical devices and implants, researchers and practitioners can create structures that are more resilient, efficient, and adaptive to the patient's body. This narrative review discusses the usage of smart materials in the field of dentistry (9).

## 2 Literature search

A comprehensive literature search was conducted using EMBASE, MEDLINE, Web of Science, Cochrane Library, and various clinical trial registries to identify relevant studies published between May 2014 to May 2024. The search was restricted to English-language publications. A combination of keywords and Boolean operators was used, including (“Four-Dimensional Printing”), (“4D Printing Technology” AND “Smart Materials”), (“Bio-Smart Materials”), and (“Shape Memory Materials” AND “Dentistry”). The initial search yielded 168 articles. After title and abstract screening, 92 articles were excluded due to duplication, irrelevance, or lack of direct focus on 4D printing in dentistry. A full-text review of the remaining 76 articles led to the exclusion of 22 studies that lacked experimental validation, had insufficient data, or focused solely on traditional 3D printing without discussing 4D transformations. Additionally, reference lists of selected articles were manually screened to ensure comprehensive coverage of relevant literature. Ultimately, 54 high-quality peer-reviewed studies were included in this review.

## 3 The core elements of 4D printing

The five essential components of 4D printing technology include the printing technique, additive manufacturing medium, stimulus, interaction mechanism, and modeling approach. The first component, the printing technique, involves the use of additive manufacturing (AM) methods to fabricate materials based on computer-generated digital data. A variety of AM techniques are employed in 4D printing, including Fused Deposition Modeling (FDM) for fabricating solid-based patterns, and Selective Laser Sintering (SLS) and Selective Laser Melting (SLM) for creating powder-based structures. Additional methods, such as Stereolithography (SLA), Direct Ink Writing, Electron Beam Melting, and Jet 3D Printing, are capable of producing 4D materials, provided the printing medium is compatible with the specific printer technology (2).

**Figure 1:**
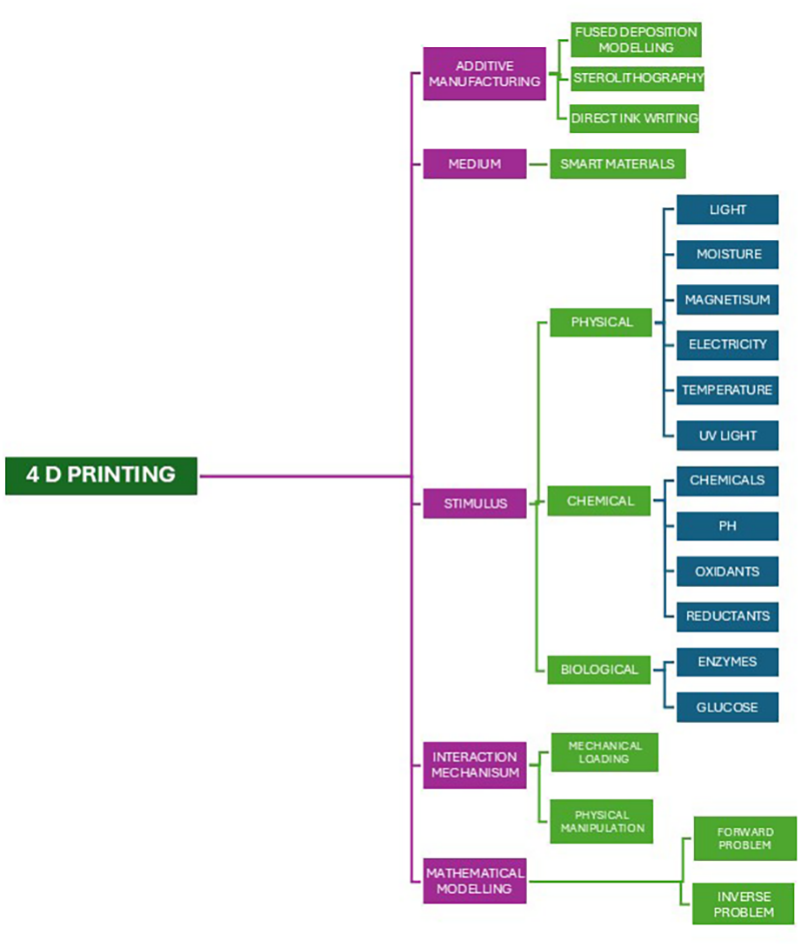
Depicts the five key aspects in 4D printing.

The subsequent phase in 4D printing involves the layer-by-layer assembly of materials designed to respond to external triggers. These materials, often termed programmable or smart materials (SMs), possess compositions that determine their responsiveness to specific stimuli, which in turn enables self-transformation. The third key component in 4D printing is the stimulus, which can be classified into biological, chemical, and physical types as depicted in Figure 1. Structural transformations in these materials, triggered by various stimuli, can induce phase changes, resulting in physical or chemical modifications such as stress relaxation and molecular movement. The final components—interaction mechanisms and mathematical modeling—are essential for predicting and controlling these responsive behaviors (10, 11). The application of stimuli in 4D printing must follow a specific sequence and duration, known as the interaction mechanism. Mathematical modeling is crucial for predicting shape evolution post-printing and for preventing structural collisions during self-assembly. 4D printing modeling can be divided into two types: the forward problem, which predicts the target shape based on material and stimulus characteristics, and the inverse problem, which determines the necessary material structure or print paths to achieve a desired shape (10).

## 4 Bio-smart materials in 4D printing

**Figure 2:**
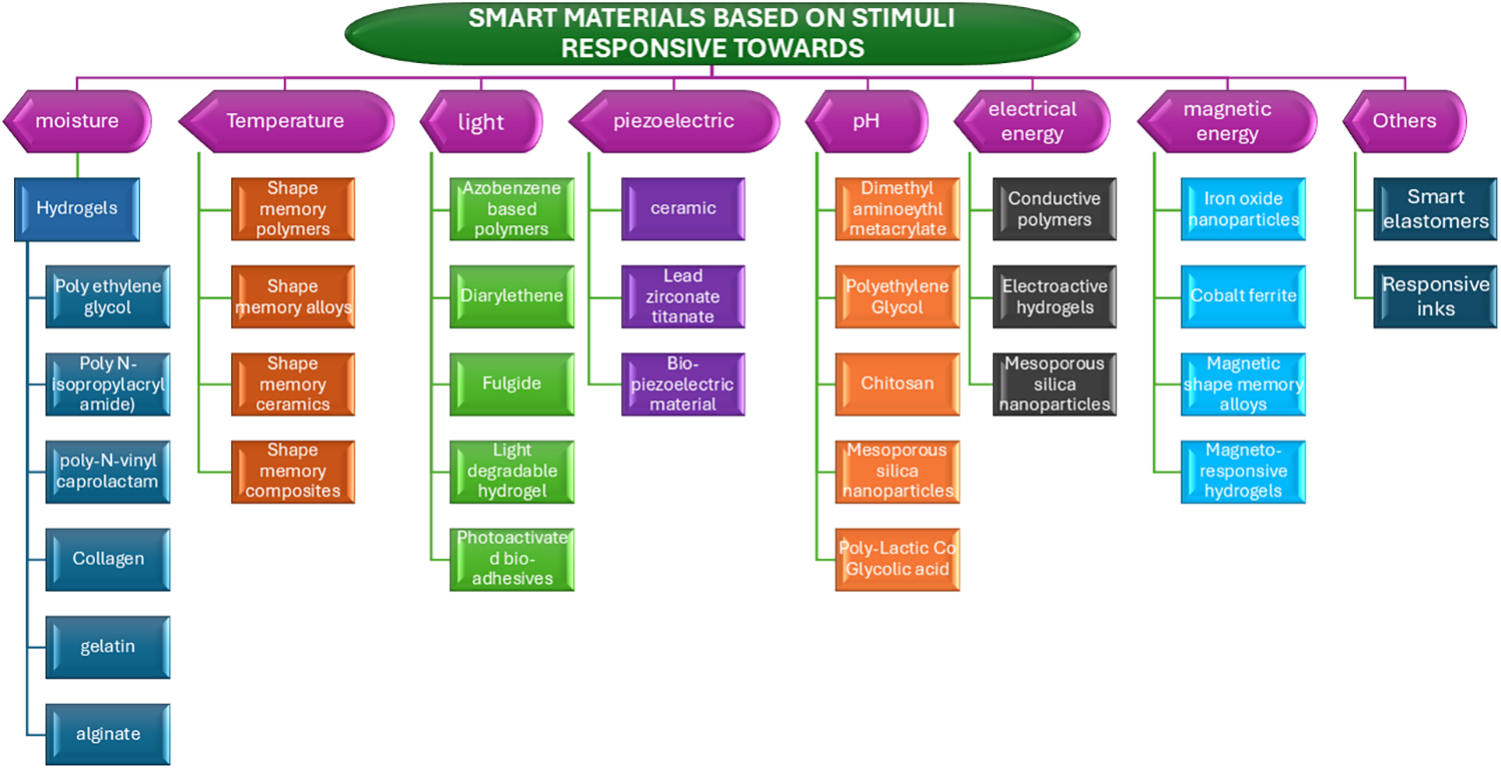
Represents the classification of Bio-smart materials based on the stimuli.

Smart materials, frequently employed in 4D printing, have the unique capability to change their properties over time. These materials respond to external stimuli and exhibit various functionalities, including self-assembly, self-healing, shape memory, adaptive capacity, and color changes under UV or visible light. A comprehensive understanding of smart materials requires an examination of how various external factors can induce specific transformations within these materials (12). To facilitate in-depth analysis, we have categorized bio-smart materials based on their stimuli, as illustrated in Figure 2.

### 4.1 Biosmart moisture-responsive hydrogels

Adaptive materials, such as hydrogels, exhibit properties that change in the presence of water. Hydrogels consist of a cross-linked polymer network capable of swelling up to 200% of their original volume upon water exposure due to their hydrophilic nature (13). This moisture sensitivity enables them to expand significantly, while also providing high compressive strength. Typically, hydrogels are used in hydrophilic environments, allowing moisture absorption until they reach saturation. The degree of hydrogel expansion can also be modulated by adjusting the water temperature. Moisture-sensitive hydrogels are widely applied in fields such as drug delivery, utilizing differential swelling for controlled release, and in cell encapsulation, where a protective membrane shields transplanted cells from immune reactions (14).

Hydrogels cross-linked polymer structure, which absorbs substantial water without dissolving, facilitates their ability to fold, stretch, bend, and undergo geometric expansion, making them ideal for advanced printing applications. Additionally, they are compatible with embedded bioactive compounds that support cell proliferation and differentiation. Smart hydrogels possess tunable properties, responding to external stimuli such as pH, temperature, and ion concentration (15). These characteristics enable the development of water-responsive micro-actuators and reversible origami structures through swelling, although water absorption persists until full saturation, complicating precise control in intermediate stages. Temperature regulation of the aqueous environment is one method to address this challenge. The typically slow response rate of hydrogels results from low water diffusivity (10^−10^–10^−^⁹ m^2^/s) and a low modulus (usually several hundred kPa), which necessitates considerable swelling for effective shape transformation (16).

Various polymers are used in hydrogels for 4D bioprinting, such as “poly(ethylene glycol) (PEG), poly(N-isopropylacrylamide) (PNIPAM), poly-N-vinyl caprolactam (PNVCL), collagen, gelatin, and alginate (17)”. Yuan et al. developed porous shape memory cryogel microspheres (CMS) from methacrylated gelatin (GelMA), which supported vascularized bone tissue formation when loaded with hBMSCs and HUVECs (18). Ying et al. created a nanofibrous gelatin scaffold chemically modified with heparin to enable sustained release of “bone morphogenetic protein-2”, suitable for injectable scaffolds in bone regeneration (19).

Jiang et al. fabricated collagen scaffolds that can return to their original shape upon moisture exposure, retaining properties that promote chondrocyte adhesion and growth (20). Ding et al. developed hydrogels using eight-armed PEG-acrylate and oxidized-and-methacrylated alginate, forming double-layer scaffolds capable of encapsulating multiple cell types (21).

Hu et al. incorporated chitosan into PLA scaffolds, creating microspheres that enhanced cell adhesion and alkaline phosphatase activity while slowing degradation. In osteogenesis studies, “poly(ε-caprolactone)-diacrylate (PCLDA) hydrogel bilayer films” were shown to promote mesenchymal stem cell (MSC) differentiation into osteogenic lineages. *in vivo*, these films encouraged active angiogenesis and improved bone healing, with an increase in “CD34, RUNX2, and OSX-positive cells”, indicating robust bone tissue regeneration (22).

### 4.2 Thermoresponsive smart materials

These smart materials are capable of shape transformation in response to temperature changes. During the manufacturing process, these smart materials undergo specific thermal treatments to become thermoresponsive (23). This property is particularly advantageous in restorative dentistry, where intraoral restorative materials must adapt to temperature fluctuations to minimize microleakage. Additionally, thermoresponsive materials show promise for localized drug delivery, where controlled drug release can be triggered by body temperature variations. Certain advanced materials can retain medication while circulating in the bloodstream and release it specifically at tumor sites with elevated temperatures (24). The various types of thermoresponsive smart materials include:

#### 4.2.1 Shape memory polymers (SMPs)

Shape memory polymers (SMPs) are a class of smart materials capable of controlled shape transformation in response to external stimuli, such as “light, temperature changes, magnetic fields, or mechanical forces”. For SMPs to perform these transformations, they must possess two essential features: a network structure that defines their permanent (memory) shape, and a switching segment that enables substantial changes in the polymer network's mobility, allowing for temporary shape alterations (25). Several synthesis approaches are effective in developing SMPs, including the “one-step polymerization of monomers or prepolymers with cross-linking agents, chemical cross-linking of high molecular weight thermoplastic polymers, direct blending of different polymers, and single-step synthesis of phase-segregated block copolymers” (26).

These polymers have gained significant applications in the biomedical and dental fields, where they serve as restorative materials with anticaries properties and are incorporated into composites with antibacterial functionalities. In particular, SMPs can improve the controlled release of therapeutic agents, thereby reducing the risk of opportunistic infections and limiting the progression of dental caries. Despite the considerable variations in temperature and pH within the oral environment caused by food intake, these SMPs are engineered to preserve their shape and mechanical properties, making them highly suitable for dental applications. A critical attribute of these polymers is their ability to regulate thermally induced volumetric transformations, further enhancing their performance in dynamic intraoral conditions (27).

SMPs are effectively used in smart obturation systems to prevent new caries formation. Polyurethane (PU) block copolymers, known for their hard and soft segment structures, are frequently employed in shape-memory orthodontic wires, where thermal activation at body temperature enables flexible and precise dental realignment. PU and polycaprolactone (PCL) composites, with recovery temperatures close to body temperature, are ideal for dental applications like implants due to their low degradation rates and high biocompatibility (28). Notably, PU and PCL-based SMPs exhibit antibacterial and antifungal properties, helping to reduce biofilm formation and the risk of secondary infections in implants (25). Erndt-Marino et al. developed a “photopolymerizable shape-memory foam using poly(ε-caprolactone) diacrylate (PCLDA)” that can be softened for implantation in irregular bone defects and, when polydopamine-coated, supports hydroxyapatite formation. Additionally, SMPs formulated with cyclodextrin and alginates have been explored for sustained drug release, making them promising alternatives to conventional dental materials (29).

#### 4.2.2 Shape memory alloys

Shape memory alloys (SMAs) exhibit this “shape memory effect,” reverting to a pre-defined form once heated above their transformation temperature. Another notable property of SMAs is superelasticity, which allows them to undergo significant, reversible strain during loading and unloading (30). Nickel-titanium (NiTi) alloys are an example of SMAs capable of both shape memory effect (thermal memory) and superelasticity (mechanical memory). NiTi alloys demonstrate excellent biomechanical compatibility with human bone, accommodating extension and flexion in the sagittal plane. Furthermore, additive manufacturing enables the production of NiTi devices with customized porosity, which is particularly advantageous for scaffold fabrication, as porous structures support osteoblast proliferation and bone integration (31).

Nickel-titanium SMAs are widely used in orthodontics due to their superior mechanical properties, biocompatibility, ductility, corrosion resistance, lower elastic modulus, and unique characteristics like superelasticity and shape memory effect (32). Akbarinia et al. designed bone-shaped SMA implants with stable root fixation properties. At −30°C, these implants are flexible, closed-leg cylinders that can be easily inserted into bone cavities. When warmed to body temperature, they return to a bifurcated shape, applying sufficient stress and strain to stabilize the implant and prevent rotational movement. This enhanced initial stability reduces the typical bone healing period required before load-bearing (33).

#### 4.2.3 Shape memory composites

These advanced smart materials integrate the properties of shape memory polymers or alloys with other materials to form composites that exhibit a distinct shape memory effect. Typically, they consist of a polymer matrix embedded with active components such as shape memory polymers, alloys, hydrogels, or other stimuli-responsive materials allowing the matrix to maintain structural integrity while enabling the shape memory effect to occur. PCL and PLA are among the most widely utilized materials in synthetic polymers, with the viscoelastic transition of PLA/PCL blends occurring at approximately 46°C, which is nearly 10°C lower than that of pure PLA (34).

Arabiyat et al. developed hybrid materials composed of poly(ε-caprolactone)-diacrylate (PCL-DA) and poly-L-lactic acid (PLLA). This scaffold material was demonstrated to enhance the expression of osteogenic markers, including osterix, bone morphogenetic protein-4 (BMP-4), and collagen 1α1 (COL1A1), in human mesenchymal stem cells (h-MSCs) (35). In another study, a PCL/hydroxyapatite (HA) scaffold implanted in the mandible of rabbits significantly promoted bone formation around the subperiosteal implant and improved the stability of a titanium implant (36). Liu et al. developed a material aimed at promoting bone repair in mandibular defects in rabbits by combining PCL with hydroxyapatite and coating the surface with a layer of calcium alginate and BMP-2 (37). Additionally, Yu et al. utilized polyurethane/nano-hydroxyapatite (SMPU/nHAP) composite scaffolds for minimally invasive surgery and bone repair. This porous composite scaffold significantly reduced surgical duration while promoting bone cell growth (38).

#### 4.2.4 Shape memory ceramics (SMCs)

Similar to SMAs, shape memory ceramics (SMCs) can display super elasticity, allowing them to undergo substantial deformation and recovery, or the shape memory effect, enabling a transition between predefined shapes in response to external stimuli. Certain brittle ceramics also experience martensitic transformations and exhibit shape memory effects similar to SMAs (39). Zang et al. reported that the advanced ceramic zirconia reduces inflammatory responses and plaque adhesion, inhibits microbial growth, and regulates fibroblast adhesion and proliferation. Additionally, zirconia is suitable for implant applications, promoting effective osseointegration with hard tissue (40).

### 4.3 Photo- responsive smart materials

These advanced materials respond to light exposure, which acts as an indirect stimulus by causing them to heat up. One significant advantage of using light as a stimulus is that it induces rapid reactions and long-lasting changes in the material, in contrast to moisture (13). Photosensitive materials require illumination to operate effectively, and light exposure can trigger various transformations, such as alterations in size, shape, and charge production. In pharmaceutical applications, light is utilized to facilitate drug delivery by causing capsules to rupture at specific wavelengths of radiation, thereby releasing the intended medication. Examples of photosensitive materials include azobenzene, stilbene, fulgide, diarylethene, and photosensitive metal nanostructures (41).

#### 4.3.1 Azobenzene based polymers

Photo-responsive smart polymers can be produced by functionalizing the material with photosensitive molecules, including “cinnamic acid (CA), cinnamylidene, or azo compounds”. Among these, azobenzene is the most commonly used photosensitive molecule due to its rapid response when exposed to the appropriate wavelength of light. These molecules can either be chemically bonded to a polymer or exist as free-floating entities within a matrix. Azobenzenes have also been employed as biosensors and have been shown to influence the structure and properties of peptides, nucleic acids, and antibody-antigen interactions. Due to their unique antibacterial properties, azobenzenes hold significant potential for application in dental restorative materials (42). Trivedi et al. synthesized methacrylated azobenzene nanogels and incorporated them within bisphenol A-glycidyl methacrylate (Bis-GMA) to evaluate their efficacy in reducing bacterial colonization by cariogenic Streptococcus mutans biofilms, while preserving the mechanical strength and structural integrity of the critical adhesive interface between the restoration and tooth. The azobenzene nanogels demonstrated a 66% reduction in biofilm formation and effectively maintained the mechanical properties and micro-tensile bond strength of the adhesive network (43).

#### 4.3.2 Photodegradable hydrogels

These biodegradable materials provide an alternative strategy for releasing therapeutic agents from polymeric matrices. The release can be modulated by controlling the degradation rate of the hydrogel. By tailoring their decomposition to respond to specific enzymes, hydrogels can be designed to deliver therapeutic compounds precisely at target sites within the body. Such systems are useful for drug delivery and the controlled release of growth factors (44). GelMA, a newly developed photosensitive hydrogel biomaterial, has attracted significant attention as a scaffold for its ability to mimic the three-dimensional cellular microenvironment. In a study by Pan et al., GelMA hydrogels were used to encapsulate periodontal ligament stem cells to facilitate bone regeneration (45). Fraser et al. developed a PEG hydrogel modified with peptides designed to regulate two key functions: periodontal ligament cell (PDLC) alkaline phosphatase (ALP) activity and matrix mineralization. *in vitro* experiments demonstrated that peptide-functionalized PEG hydrogels enhanced cell adhesion and promoted matrix mineralization, while *in vivo* studies indicated that these hydrogels significantly improved new bone formation (46).

#### 4.3.3 Photoactivated bioadhesives

Hydrogel-based bioadhesives offer significant potential in both soft and hard tissue engineering due to their customizable composition and physical characteristics. The ability to precisely control the microstructure, mechanical properties, and degradation rates of these hydrogels makes them promising candidates for the targeted delivery of therapeutic agents *in vivo*. Sani et al. developed a multifunctional adhesive hydrogel with antimicrobial properties for treating peri-implant disease. Rapidly crosslinked using standard dental curing systems, this hydrogel effectively bonds to both soft (gingiva) and hard tissues (dental implants and bone). It demonstrates strong adhesion, mechanical durability, antimicrobial activity, cytocompatibility, biodegradability, and promotes bone regeneration (47, 48).

### 4.4 Electro-responsive smart materials

Electro-responsive materials encompass a wide range of substances that can alter their properties in response to an electric current. Electricity serves as an indirect stimulus, generating heat as it passes through the material. This category includes conductive polymers such as polypyrrole and polyaniline, as well as shape memory polymers embedded with fillers or nanostructures that respond to electrical stimuli. These materials occasionally incorporate electrically conductive additives, such as carbon nanotubes (CNTs), graphene, and other nanoparticles (49). Okuzaki et al. created an origami robot using 4D printing with an electro-sensitive polypyrrole-based organic polymer. Currently, these bioactive materials are being explored for the development of constructs in muscle and neural tissue engineering applications. Additionally, conductive polymer-based hydrogels demonstrate excellent printability and biocompatibility, making them suitable candidates for 4D printing of biomaterials (50). Although the potential of electro-responsive smart materials in 4D printing for dental applications is promising, there is a notable scarcity of research in this area. Consequently, further investigations are necessary to explore and validate the efficacy and applications of these advanced materials in dentistry.

### 4.5 Magneto-responsive smart materials

Materials employed in 4D printing that undergo shape changes in the presence of a magnetic field are referred to as magneto-responsive materials. These materials typically consist of a polymer matrix embedded with magnetic particles, such as iron oxide or neodymium, or include magnetoresponsive fillers that react to magnetic stimuli (51).

Zhang et al. investigated the behaviour of 4D printed shape-memory structures fabricated under a magnetic field, utilizing poly(lactic acid) (PLA)-Fe₃O₄ composite filament. Their results showed that printed objects containing 15% Fe₃O₄ by weight could “rapidly recover their original shape” within seconds when exposed to a magnetic field with a frequency of 27.5 kHz (52). Zheng et al. developed biocompatible nanocomposites composed of poly-d,l-lactic acid (PDLLA) and Fe₃O₄ and evaluated their shape-memory performance under a 20 kHz alternating magnetic field. The results demonstrated an exceptionally high shape recovery ratio (52). Zhao and colleagues created shape-memory PLA-FeO₄ composites to be used as scaffolds for bone tissue. According to their research, these scaffolds may be efficiently triggered by magnetic fields, which greatly increases cell adhesion (53).

### 4.6 Piezo-electric smart materials

Piezoelectric materials generate electric current when subjected to mechanical stress, a phenomenon referred to as piezoelectricity. This characteristic enables piezoelectric materials to undergo shape changes in response to mechanical forces, which may render them particularly useful in 4D printing applications. Notably, even minimal mechanical stresses and electric charges can induce significant structural modifications in these materials (13).

Piezoelectric materials have broad applicability, particularly in the medical field, such as in implant technology. By selectively applying electrical stimulation to piezoelectric-active implants, the rate of bone formation can be accelerated, while bone resorption is reduced which can substantially improve the osseointegration of the implant (54). Bio-piezoelectric materials have garnered considerable attention for their potential in bone repair by mimicking the electrical microenvironment (EM) of native tissue. Scaffolds made from bio-piezoelectric materials using 4D printing technology can dynamically alter their functionality over time, creating a programmable tissue EM that responds to external stimuli and facilitates bone regeneration (54).

### 4.7 pH responsive smart materials

These are sophisticated materials whose form and volume may change in response to changes in pH (13). pH-responsive nanocarriers are among the most widely used systems for drug delivery within the oral cavity. These nanocarriers are designed with functional groups, “such as amines or acid-sensitive bonds”, that enable them to respond to pH fluctuations. When exposed to changes in pH, these bonds undergo protonation, deprotonation, or cleavage, triggering the controlled release of the medication (13). Yao et al. developed pH-responsive microspheres using chitosan. Their results indicated that drug release occurred exclusively in acidic environments, suggesting that these microspheres have potential as carriers for intelligent drug delivery systems. Yu et al. created drug-loaded microparticles composed of chitosan, alginates, and pectin. These microparticles exhibited high pH sensitivity, making them suitable for site-specific protein drug delivery via oral administration (55).

## 5 Other smart materials

### 5.1 Smart elastomer

Shape memory elastomers (SMEs) are elastic polymer networks and represent an emerging class of active elastomers with dual- or multi-shape capabilities. Typically, SMEs display two structural states: an original (permanent) shape and a deformed (temporary) shape, which can reversibly transition under specific environmental conditions. Compared to shape memory polymers (SMPs), SMEs are softer and more elastomeric, as elastomers are characterized by their inherent “elasticity,” low tensile strength, and high elongation at break. This allows them to endure significant elastic deformation without rupture. Additionally, elastomers exhibit relative softness and deformability at ambient temperatures due to their low glass transition temperatures (Tg) (56).

### 5.2 Responsive inks

Responsive inks used in 4D printing technology are specialized materials designed with dynamic properties that respond to external stimuli. These inks are engineered to undergo specific transformations, including changes in color, shape, conductivity, or mechanical properties, in response to environmental triggers such as temperature fluctuations, light exposure, humidity variations, and chemical interactions as depicted in Table 1. Responsive inks, typically formulated with functional components such as smart polymers, nanoparticles, or reactive dyes dispersed within a carrier medium like a polymer solution or solvent, enable the creation of objects with time-evolving, programmable behaviors. In 4D printing, these inks support the fabrication of complex structures whose responsive properties are pre-engineered in the ink composition and can be activated by external stimuli during the printing process (15). Miao et al. utilized 4D bioprinting to develop nerve guide conduits (NGCs) using a bio-ink containing graphene. A photosensitive polymer induced bending in the printed structure, while graphene-based nanoparticles enhanced the biomaterial's conductivity, facilitating the differentiation of human mesenchymal stem cells (hMSCs) into neural cells (57). Gladman et al. incorporated a plant-inspired hydrogel composite bio-ink, consisting of acrylamide matrices and cellulose fibrils, to create a dynamic, biomimetic 4D-printed structure. This design promotes an increase in pore size, which in turn enhances the supply of oxygen and nutrients to the internal regions of the scaffolds (58).

**Table 1:** Represents various types of 4D printing responsive inks and its application in dentistry.

Types of responsive inks	Inference
Thermochromic inks	These inks exhibit color changes in response to temperature variations and can be integrated into dental appliances, such as mouthguards or orthodontic aligners (58).
Hydrochromic inks	These materials change their color or transparency in response to moisture exposure, making them suitable for the development of dental devices that offer visual feedback on oral hygiene or moisture levels (59).
pH sensitive inks	These inks exhibit color changes in response to variations in pH levels and can be incorporated into dental materials, such as adhesives or restorative materials, to aid in the prevention of dental caries (58).
Antimicrobial inks	These inks are formulated with agents that inhibit bacterial growth and support oral health. They can be utilized in the fabrication of dental implants or prosthetic appliances to enhance healing and promote tissue regeneration (59).
Smart polymer inks	These materials can undergo reversible changes in shape, stiffness, or conductivity in response to external stimuli such as temperature, light, or moisture. They are suitable for the fabrication of dental devices, including shape-changing orthodontic appliances or dental materials that adapt to dynamic changes in the oral environment (59).

## 6 Conclusion

4D printing represents a transformative advancement in the field of dentistry, offering innovative solutions through the development of smart devices and adaptive dental materials. By utilizing intelligent materials, 4D printing creates products capable of adjusting to environmental changes, enhancing flexibility and functionality. It enables the customization of dental prosthetics, orthodontic devices, and regenerative solutions, benefiting both healthcare providers and patients. Personalized 4D implants, instruments, and devices not only reduce surgical and recovery times but also improve clinical outcomes and implant success rates. Although still under research and development this emerging technology holds immense potential in dentistry, it promises to address future challenges in dentistry, paving the way for more efficient, patient-specific care.
